# Qualitative and Quantitative Analysis of Phenolic Acids, Flavonoids and Iridoid Glycosides in Yinhua Kanggan Tablet by UPLC-QqQ-MS/MS

**DOI:** 10.3390/molecules200712209

**Published:** 2015-07-03

**Authors:** Yu Lin, Wen Xu, Mingqing Huang, Wei Xu, Huang Li, Miao Ye, Xun Zhang, Kedan Chu

**Affiliations:** College of Pharmacy, Fujian University of Traditional Chinese Medicine, Fuzhou 350122, China; E-Mails: xwfjlab@163.com (W.X.); huangmingqing3413@gmail.com (M.H.); xwfjtcm@sina.com (W.X.); lihuang3413@gmail.com (H.L.); ymfjtcm@163.com (M.Y.); zxfjtcm@gmail.com (X.Z.); chukedan@gmail.com (K.C.)

**Keywords:** Yinhua Kanggan tablet, ultra-performance liquid chromatography, triple quadrupole mass spectrometry, quality control

## Abstract

A simple, rapid and specific ultra-performance liquid chromatography-triple quadrupole mass spectrometry method was developed for the analysis of 29 bioactive components (10 phenolic acids, 16 flavonoids, and three iridoid glycosides) in Yinhua Kanggan tablet (YHKGT), a herbal prescription used for treating upper respiratory infections, fevers, coughs and pharyngalgia. The separation was successfully achieved using a Waters Cortecs UPLC C18 column (50 × 2.1 mm, 1.6 μm) and gradient elution with water-0.1% formic acid and acetonitrile. Polarity switching mode was used in the optimization of multiple reaction monitoring conditions. The analytical method was validated for linearity, precision and accuracy. Calibration curves for the 29 marker compounds showed good linear regression (*r* > 0.9982). The limits of detection (LOD) and limits of quantification (LOQ) for the 29 analytes were in the range of 0.03–4.99 ng/mL and 0.16–14.87 ng/mL, respectively. The relative standard deviation (RSD) values of intra-day precision, inter-day precision, repeatability, and stability were less than 2.79%, 4.87%, 4.18% and 4.71%, respectively. The recoveries of the 29 marker compounds were in the range of 94.67%–104.78% (RSD ≤ 4.72%). These results have shown that this developed method was efficient for the quality evaluation of YHKGT.

## 1. Introduction

Recently, herbal medicines have received great interest and have been used as an important part of health care in the anti-viral treatment field since they have relatively few side-effects compared to modern therapeutics [1]. Yinhua Kanggan tablet (YHKGT), a well-known traditional Chinese medicinal preparation, containing *Lonicera japonica* (Jinyinhua), *Cyrtomium fortunei* (Guanzhong), *Melicope pteleifolia* (Sanchaku), *Vitex negundo var. cannabifolia* (Mujinggen), *Tadehagi triquetrum* (Hulucha), and *Mussaenda pubescens var. alba* (Shangancao), has been widely used in China to treat upper respiratory infections, fevers, coughs and pharyngalgia [2]. Previous studies have indicated that three types of compounds, including phenolic acids, flavonoids and iridoids, are responsible for the overall therapeutic effects of Jinyinhua as well as Mujinggen [3,4,5,6,7,8,9]. However, relatively little research has been done in regard to the chemical constituents and pharmacological effects of the other herbal ingredients in YHKGT [10,11,12].

Although herbal medicines are increasingly being understood and accepted by more and more people around the world, the problem of quality control remains one of the major obstacles for their internationalization. A published paper has described a high performance liquid chromatography-diode-array detector (HPLC-DAD) method for the determination of the content of chlorogenic acid, one of the major components in YHKGT [13], but the efficacy of YHKGT should be associated with the synergistic or interactive action of various types of compounds derived from its component herbs rather than only one of them. Actually, the present quality control method severely restricts the clinical applications and in-depth study of YHKGT, therefore, it is of great significance to develop a more sensitive and efficient analytical method for the determination of more bioactive components in YHKGT for its quality assurance. Moreover, the aforementioned three types of active compounds of YHKGT should be selected for the quality control analysis.

In the present study, we have developed and validated for the first time a polarity switching ultra-performance liquid chromatography coupled with triple quadrupole mass spectrometry (UPLC-QqQ-MS) method for the rapid simultaneous determination of 29 active components (10 phenolic acids, 16 flavonoids and three iridoid glycosides) in YHKGT. Thirteen batches of YHKGT were collected for the analysis. Additionally, to ensure the accuracy and the sensitivity of quantification, 2-hydroxycinnamic acid, liquiritin, and albiflorin were employed as internal standards for phenolic acids, flavonoids, and iridoids, respectively.

## 2. Results and Discussion

### 2.1. Optimization of Sample Preparation

In order to achieve optimal extraction efficiency, the variables involved in the extraction, such as extract solvent and extract method, were optimized. Due to the different polarity and water-solubility of some analytes, 70% aqueous methanol was chosen as the extraction solvent. To find the best extraction method, ultrasonic extraction, refluxing and Soxhlet extraction were selected because of their relatively shorter extraction time than percolation and maceration. The results suggested that ultrasonic extraction was simpler and more efficient than refluxing and Soxhlet extraction in extracting typical compounds **1**–**29** using 70% aqueous methanol as extraction solvent (Figure S1). Moreover, to obtain the optimal extraction efficiency, the effects of different factors including the different concentrations of methanol-water solution (30%, 50%, 70% and 90%), solvent volume (10, 50, 100 and 150 times) and extraction time (15, 30, 45 and 60 min) on the extraction performance were evaluated. An almost equal amount of sample (0.2 g) was extracted and analyzed using the described procedure. As a result, compared with the extraction yields of different factors of the extraction solution, volume and extraction time for 29 typical compounds, it was found that ultrasonic extraction with 100 times the volume of 70% methanol–water for 30 min for one time was an optimum method to prepare the sample solution (Figures S2–S4 ).

### 2.2. Optimization of Chromatographic Conditions

The chromatographic conditions were optimized to improve the resolution and sensitivity and shorten the analysis time. Different mobile phases including methanol-water and acetonitrile-water were examined. Acetonitrile-water was found to produce better peak shapes than methanol-water. Interestingly, using 5% methanol to water could increase the resolution of several isomers such as schaftoside and isoschaftoside. Moreover, it was found that formic acid was not only beneficial to improving the chromatographic separation, but also in improving the ionization efficiency of analytes. In addition, due to their similar structures, retention time and ionization response in the negative ion mode, 2-hydroxycinnamic acid, liquiritin and albiflorin were chosen as internal standards for phenolic acids, flavonoids and iridoids, respectively.

**Figure 1:**
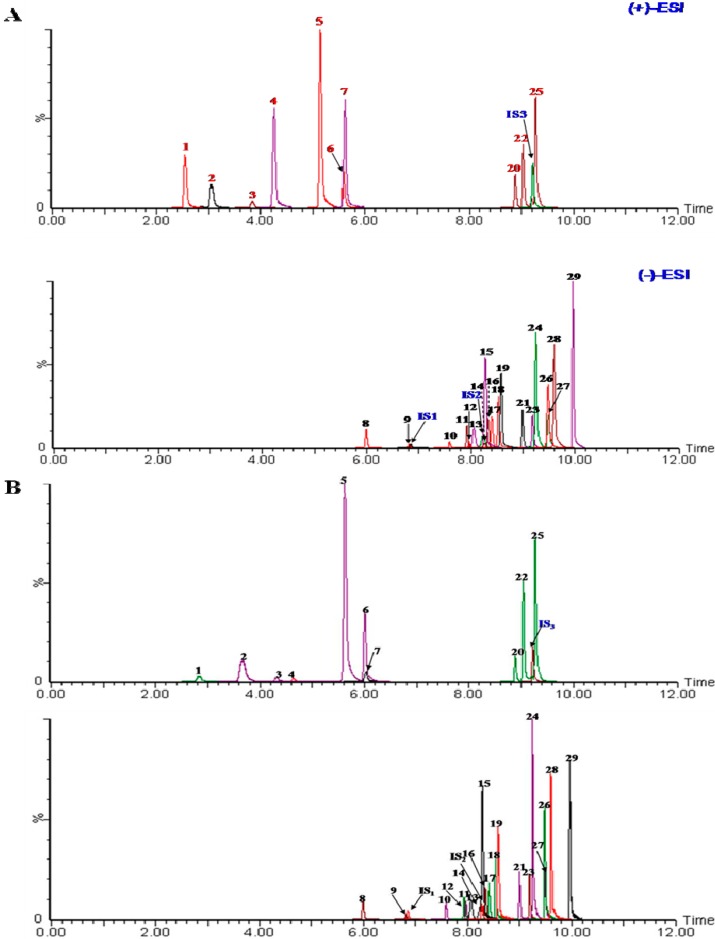
LC-MS/MS MRM chromatogram of 29 target standards(included 3 internal standards) (**A**) and samples (**B**): (**1**) protocatechuic acid; (**2**) neochlorogenic acid; (**3**) protocatechualdehyde; (**4**) 4-hydroxybenzoic acid; (**5**) chlorogenic acid; (**6**) cryptochlorogenin acid; (**7**) caffeic acid; (**8**) swertiamarin; (**9**) sweroside; (**10**) schaftoside; (**11**) agnuside; (**12**) isoschaftoside; (**13**) flavosativaside; (**14**) vitexin 2′′-rhamnoside; (**15**) rutin; (**16**) vitexin; (**17**) hyperoside; (**18**) isoquercitrin; (**19**) luteoloside; (**20**) isochlorogenic acid B; (**21**) kaempferol-3-*O*-rutinoside; (**22**) isochlorogenic acid A; (**23**) astragalin; (**24**) apigenin-7-glucoside; (**25**) isochlorogenic acid C; (**26**) luteolin; (**27**) quercetin; (**28**) apigenin; (**29**) casticin; (internal standard 1, IS_1_) albiflorin; (**IS_2_**) liquiritin; (**IS_3_**) 2-hydroxycinnamic acid.

In order to develop a sensitive and accurate quantitative method, the MS/MS fragmentation for each analyte was investigated by direct infusion of the single standard solution into the mass spectrometer (−)-ESI and (+)-ESI source to optimize MS parameters, including product ion, cone voltage, and collision energy, the product ion of each analytes. The negative ion mode was found to be more suitable for flavonoids and iridoid glycosides analyses, while positive ion mode was found to be more suitable for phenolic acids. Therefore, ion polarity switching mode was used in the optimization of MRM conditions in the quantitative analysis. The optimum results are shown in Table 1 and the MRM chromatograms of the 29 markers are shown in Figure 1A. LC/MS chromatograms of analytes from a real sample is presented in Figure 1B.

**Table 1:** Retention time, related MS data of 29 investigated compounds and three internal standards in the UPLC-QqQ MS analysis.

Compounds	t_R_ (min)	Precursor Ion (*m/z*)	Product Ion (*m/z*)	Cone Voltage (V)	Collision Energy (eV)	Polarity
Protocatechuic acid	2.52	155	93	25	12	Positive
Neochlorogenic acid	3.07	355	163	20	18	Positive
Protocatechualdehyde	3.85	139	93	25	15	Positive
*p*-Hydroxybenzoic acid	4.25	139	121	15	10	Positive
Chlorogenic acid	5.15	355	163	20	18	Positive
Cryptochlorogenin acid	5.61	355	163	20	18	Positive
Caffeic acid	5.63	181	163	20	12	Positive
Swertiamarin	5.99	419	179	20	12	Negative
Sweroside	6.82	403	179	25	12	Negative
Schaftoside	7.6	563	443	30	28	Negative
Agnuside	7.93	465	285	50	22	Negative
Isoschaftoside	7.97	563	443	30	28	Negative
Flavosativaside	8.00	593	413	50	22	Negative
Vitexin 2′′-rhamnoside	8.28	577	413	40	25	Negative
Rutin	8.33	609	300	35	35	Negative
Vitexin	8.35	431	311	45	20	Negative
Hyperoside	8.42	463	300	45	28	Negative
Isoquercitrin	8.53	463	300	45	28	Negative
Luteoloside	8.58	447	285	50	28	Negative
Isochlorogenic acid B	8.88	517	163	20	22	Positive
Kaempferol-3-*O*-rutinoside	8.99	593	285	35	30	Negative
Isochlorogenic acid A	9.04	517	163	20	22	Positive
Astragalin	9.19	447	285	35	22	Negative
Apigenin-7-glucoside	9.25	431	267	55	35	Negative
Isochlorogenic acid C	9.26	517	163	20	22	Positive
Luteolin	9.48	285	133	50	32	Negative
Quercetin	9.53	301	151	40	25	Negative
Apigenin	9.59	269	151	45	32	Negative
Casticin	9.97	373	343	35	22	Negative
Albiflorin (IS1)	6.85	525	121	25	25	Negative
Liquiritin (IS2)	8.28	417	255	25	20	Negative
2-Hydroxycinnamic acid (IS3)	9.22	165	123	15	12	Positive

### 2.3. Identification of Compounds with UPLC-MS/MS

The established analytical method was applied to identify the 29 compounds in YHKGT. The structures were unambiguously assigned based on their retention times and MS spectra of the reference standards (Table S1, Supplementary Information). The ESI mass spectra gave characteristic quasi-molecular ions of protocatechuic acid ([M + H]^+^ ion at *m*/*z* 155), neochlorogenic acid ([M + H]^+^ ion at *m*/*z* 355), protocatechualdehyde ([M + H]^+^ ion at *m*/*z* 139), *p*-hydroxybenzoic acid ([M + H]^+^ ion at *m*/*z* 139), chlorogenic acid ([M + H]+ ion at *m*/*z* 355), cryptochlorogenin acid ([M + H]^+^ ion at *m*/*z* 355), caffeic acid ([M + H]^+^ ion at *m*/*z* 181), swertiamarin ([M − H + HCOOH]^−^ ion at *m*/*z* 419), sweroside ([M − H + HCOOH]^−^ ion at *m*/*z* 403), schaftoside ([M − H]^−^ ion at *m*/*z* 563), agnuside ([M − H]^−^ ion at *m*/*z* 465), isoschaftoside ([M − H]^−^ ion at *m*/*z* 563), flavosativaside ([M − H]^−^ ion at *m*/*z* 593), vitexin 2′′-rhamnoside ([M − H]^−^ ion at *m*/*z* 577), rutin ([M − H]^−^ ion at *m*/*z* 609), vitexin ([M − H]^−^ ion at *m*/*z* 431), hyperoside ([M − H]^−^ ion at *m*/*z* 463), isoquercitrin ([M − H]^−^ ion at *m*/*z* 463), luteoloside ([M − H]^−^ ion at *m/z* 447), isochlorogenic acid B ([M + H]^+^ ion at *m*/*z* 517), kaempferol-3-O-rutinoside ([M − H]^−^ ion at *m/z* 593), isochlorogenic acid A ([M + H]^+^ ion at *m*/*z* 517), astragalin ([M−H]^−^ ion at *m*/*z* 447), apigenin-7-glucoside ([M − H]^−^ ion at *m*/*z* 431), isochlorogenic acid C ([M + H]^+^ ion at *m*/*z* 517), luteolin ([M − H]^−^ ion at *m*/*z* 285), quercetin ([M − H]^−^ ion at m/z 301), apigenin ([M − H]^−^ ion at *m*/*z* 267), and casticin ([M − H]^−^ ion at *m*/*z* 373).

**Figure 2:**
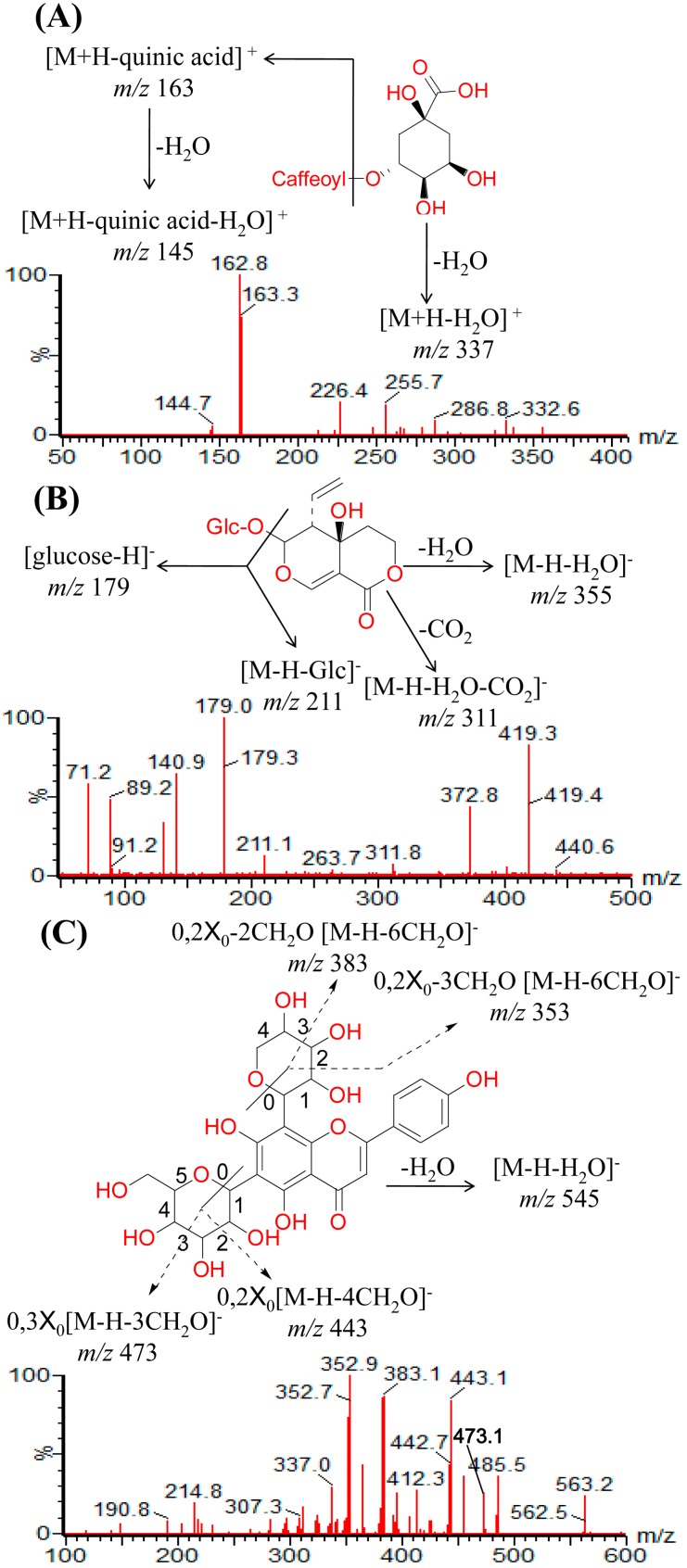

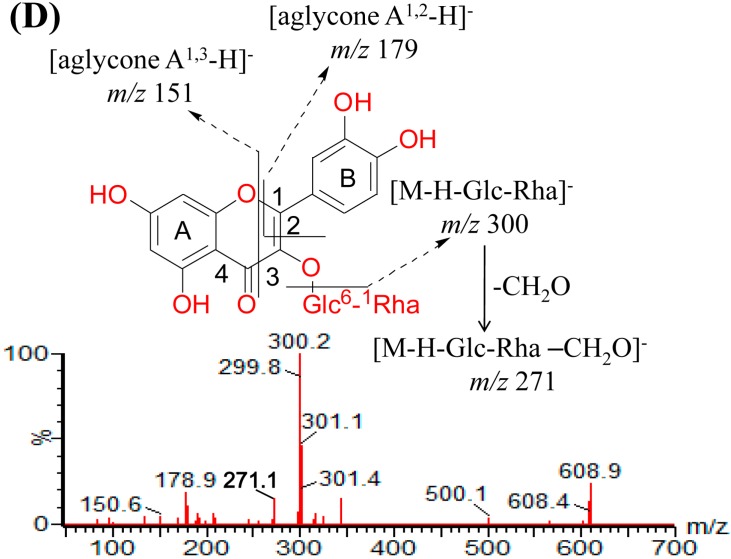
The ESI-MS/MS spectra and the proposed fragmentation pathway of chlorogenic acid (**A**); swertiamarin (**B**); schaftoside (**C**); and rutin (**D**).

Moreover, MS/MS analysis gave characteristic fragmentation behavior of the 29 compounds identical to previous reports [14,15,16,17,18,19,20,21,22,23,24,25,26]: *m*/*z* 137→111→93 for protocatechuic acid, *m*/*z* 337→163→145 for neochlorogenic acid, *m*/*z* 121→111→93 for protocatechualdehyde, *m*/*z* 121→95→77 for *p*-hydroxybenzoic acid, *m*/*z* 337→163→145 for chlorogenic acid, *m*/*z* 337→163→135 for crypto-chlorogenin acid, *m*/*z* 163→135→123 for caffeic acid, *m*/*z* 355→311→211→179 for swertiamarin, *m*/*z* 195→179→125 for sweroside, *m*/*z* 545→473→443→383→353 for schaftoside, *m*/*z* 303→285→165→137 for agnuside, *m*/*z* 473→443→383→353 for isoschaftoside, *m*/*z* 473→413→293 for flavosativaside, *m*/*z* 559→547→413→293 for vitexin 2′′-rhamnoside, *m*/*z* 300→271→179→151 for rutin, *m*/*z* 341→323→311→283 for vitexin, *m*/*z* 300→271→179→151 for hyperoside, *m*/*z* 445→401→300→271→179 for isoquercitrin, *m*/*z* 429→383→285 for luteoloside, *m*/*z* 499→475→429→163 for isochlorogenic acid B, *m*/*z* 535→285→255→151 for kaempferol-3-*O*-rutinoside, *m*/*z* 499→429→163 for isochlorogenic acid A, m/z 417→285→239→151 for astragalin, *m*/*z* 267→239→151→117 for apigenin-7-glucoside, *m*/*z* 499→429→163 for isochlorogenic acid C, *m*/*z* 237→175→151→133 for luteolin, *m*/*z* 273→255→151→133 for quercetin, *m*/*z* 225→151→117 for apigenin, and *m*/*z* 358→343→328→300 for casticin. Chlorogenic acid, rutin, schaftoside, and swertiamarin are used as examples to clarify the detailed identification processes of phenolic acids, *O*-glycosyl flavonoids, *C*-glycosyl flavonoids, and iridoid glycosides in Figure 2A–D, respectively.

### 2.4. Method Validation

**Figure 3:**
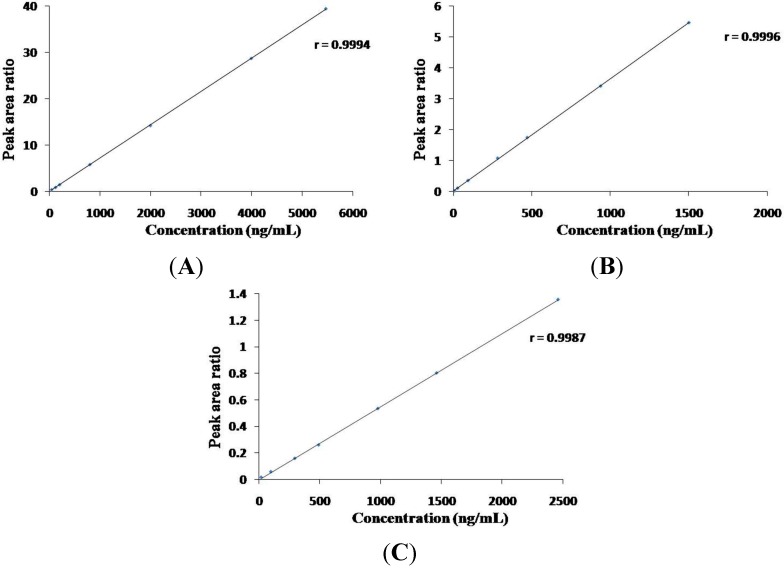
Linearity of representative compounds (chlorogenic acid (**A**); swertiamarin (**B**) and schaftoside (**C**)).

(a) *Linearity*. The calibration curves, plotted with at least six concentrations of standard solutions, were constructed from the peak areas ratios of each standard to IS *vs.* concentration of each analyte. Acceptable linear correlation at these conditions was confirmed by correlation coefficients (*r*, 0.9987–0.9998) (Figure 3).

(b) *LOD and LOQ*. Limits of detection (LODs) and quantification (LOQs) are three times and ten times the noise level, respectively. For each target compound, the LODs and LOQs were determined by serial dilution of standard solution under the described UPLC-QqQ MS conditions. The LODs (S/N = 3) and LOQs (S/N = 10) for all standard analytes were in the range of 0.03–4.99 and 0.16–14.87 ng/mL, respectively, indicating that this method is sensitive for the quantitative determination of major components in YHKGT samples (Table S2, Supplementary Information).

(c) *Precision*. Intra- and inter-day variations were chosen to determine the precision from standard solutions the developed method. For intra-day precision test, the standards solutions were analyzed for six replicates within 1 day, while for inter-day precision test, the solutions were examined in duplicates for consecutive 3 days. The RSD values of intra- and inter-day precision were in the range of 0.84%–2.79% and 1.07%–4.87%, respectively.

To confirm the precision from real samples (YHKGT), six samples of YHKGT (No. 1301014) were extracted and analyzed on three separate days. The RSD values of 29 standards were within the range of 2.03%–4.18%. In order to investigate the stability of the samples, each sample solution was analyzed within 24 h (0, 8, 12 and 24 h) at room temperature. The RSD values of the 29 analytes were all less than 4.71% within 24 h.

(d) *Stability*. Meanwhile, the stability of the standards was also investigated at 25 °C standards were analyzed every 4 h within 12 h in triplicate. In conclusion, this developed method had good precision, repeatability and stability (Table 2).

**Table 2:** Precision, repeatability and stability of the 29 investigated compounds.

Compounds	Precision from Standard Solutions (RSD, %, *n* = 6)		Precision from Real Samples (YHKGT) (RSD, %, *n* = 6)	Stability (%)	Concentration (ng/mL)
	Intra-day	Inter-day			
Protocatechuic acid	1.67	3.38	2.83	97.78 ± 3.39	399.52
Neochlorogenic acid	1.29	3.21	2.27	102.45 ± 3.03	998.16
Protocatechualdehyde	2.79	4.65	2.78	99.83 ± 3.01	39.95
*p*-Hydroxybenzoic acid	1.56	2.63	2.03	98.9 ± 2.95	199.45
Chlorogenic acid	1.70	3.55	2.34	98.34 ± 2.14	5992.3
Cryptochlorogenin acid	2.01	3.98	2.76	103.12 ± 3.84	1996.54
Caffeic acid	2.66	3.97	2.51	101.32 ± 3.94	39.95
Swertiamarin	0.84	1.07	2.80	99.02 ± 1.92	19.97
Sweroside	2.08	3.54	2.58	95.76 ± 3.37	998.6
Schaftoside	1.34	1.99	3.63	98.37 ± 2.68	1996.22
Agnuside	1.92	3.20	2.87	101.56 ± 3.04	599.36
Isoschaftoside	1.12	2.54	2.67	98.45 ± 2.43	399.31
Flavosativaside	1.01	1.63	2.08	102.89 ± 3.29	19.22
Vitexin 2′′-rhamnoside	2.20	2.41	3.03	99.43 ± 2.66	19.67
Rutin	0.87	1.51	2.33	102.36 ± 2.02	399.36
Vitexin	1.94	2.75	2.54	99.43 ± 2.39	27.42
Hyperoside	2.36	3.44	3.73	99.95 ± 3.27	50.13
Isoquercitrin	1.23	2.42	4.18	95.72 ± 2.56	297.21
Luteoloside	2.35	3.68	2.48	101.23 ± 4.27	195.87
Isochlorogenic acid B	2.14	3.06	2.19	98.45 ± 3.67	998.82
Kaempferol-3-*O*-rutinoside	1.94	3.37	4.10	99.59 ± 3.64	99.99
Isochlorogenic acid A	2.56	4.87	2.99	96.31 ± 4.54	1997.97
Astragalin	2.30	3.37	2.68	99.56 ± 2.76	99.1
Apigenin-7-glucoside	1.86	2.89	3.16	103.45 ± 3.08	9.71
Isochlorogenic acid C	1.22	3.90	2.93	96.34 ± 3.45	1996.38
Luteolin	1.30	2.34	2.64	102.62 ± 3.25	28.25
Quercetin	2.42	4.58	3.18	97.34 ± 3.91	196.3
Apigenin	1.25	1.95	2.70	98.99 ± 2.53	9.61
Casticin	2.04	3.76	2.99	101.25 ± 3.29	27.21

(e) *Accuracy*. The recovery was used to evaluate the accuracy of the method and determine by adding the mixed standard solutions with three different concentration levels (low, medium and high) to the known amounts of YHKGT sample. Triplicate experiments were conducted at each level. The percentage recoveries were calculated according to the following equation: (detected amount − original amount) × 100%/spiked amount. As shown in Table 3, the recovery rate of 29 standards varied from 94.67 to 104.78% (RSDs ≤ 4.72%), revealing the acceptable recovery and accuracy of this method.

**Table 3:** Recovery data of the proposed method (*n* = 3).

Compounds	Original (μg)	Spiked (μg)	Detected (μg)	Recovery (%)	RSD (%)
Protocatechuic acid	17.36	31.2	49.97	104.52	3.59
		62.4	81.83	103.32	2.33
		156	180.13	104.34	2.78
Neochlorogenic acid	60.09	29.7	89.60	99.36	3.48
		59.4	122.32	104.76	3.13
		148.5	215.69	104.78	2.55
Protocatechualdehyde	4.27	29.9	35.07	103.01	3.97
		59.81	67.55	104.63	2.56
		149.52	150.14	97.56	3.08
*p*-Hydroxybenzoic acid	12.47	27.6	40.14	100.25	3.86
		55.2	66.52	97.92	2.95
		138	155.98	103.99	2.90
Chlorogenic acid	365.29	29.85	395.29	100.50	3.40
		59.7	426.84	103.10	2.46
		149.24	514.22	99.79	2.14
Cryptochlorogenin acid	87.22	29.47	117.71	103.46	2.87
		58.94	148.96	104.75	3.73
		147.36	240.44	103.98	2.22
Caffeic acid	5.80	29.94	34.81	96.89	3.66
		59.89	63.03	96.89	2.87
		149.72	159.54	102.69	2.86
Swertiamarin	1.26	28.48	28.32	97.82	4.27
		56.96	57.10	98.03	2.00
		142.4	146.52	102.01	2.35
Sweroside	61.19	29.38	89.31	97.07	2.11
		58.75	120.47	100.90	2.34
		146.88	212.87	103.27	3.88
Schaftoside	85.0	29.7	116.03	101.11	2.40
		59.39	144.73	100.57	3.49
		148.48	232.67	99.45	2.84
Agnuside	30.10	29.94	58.91	96.23	3.12
		59.88	88.32	97.23	2.35
		149.7	186.40	103.74	2.33
Isoschaftoside	16.07	29.63	45.34	98.79	4.02
		59.26	72.80	97.42	2.47
		148.16	170.07	103.94	3.43
Flavosativaside	2.47	28.89	30.06	98.96	2.76
		57.78	58.70	97.32	2.50
		144.44	150.82	102.71	3.06
Vitexin 2′′-rhamnoside	3.13	29.57	33.60	103.04	2.37
		59.14	62.91	101.08	3.10
		147.84	151.41	100.30	2.95
Rutin	17.87	30.07	48.32	101.26	2.21
		60.14	79.54	102.54	3.73
		150.36	173.59	103.56	4.07
Vitexin	1.07	41.22	43.04	101.82	3.44
		82.43	85.03	101.86	2.66
		206.08	215.73	104.16	3.96
Hyperoside	2.49	30.10	30.70	103.69	3.70
		60.19	60.32	101.06	2.60
		150.48	151.61	100.43	3.93
Isoquercitrin	12.12	29.81	42.54	102.05	4.72
		59.62	71.53	99.65	3.34
		149.05	162.91	101.17	3.63
Luteoloside	7.53	29.44	36.52	98.47	4.24
		58.88	68.42	103.41	4.59
		147.2	160.87	103.90	3.04
Isochlorogenic acid B	65.22	27.72	93.59	102.34	4.37
		55.44	119.99	98.79	2.84
		138.6	208.53	103.40	2.58
Kaempferol-3-*O*-rutinoside	17.87	30.07	48.32	101.26	3.21
		60.14	79.54	102.54	2.73
		150.36	173.59	103.56	3.07
Isochlorogenic acid A	105.61	29.57	135.18	100.00	2.41
		59.14	165.23	100.81	3.07
		147.84	253.38	99.95	2.19
Astragalin	3.33	29.76	33.89	102.69	2.45
		59.52	65.60	103.88	2.86
		148.8	159.29	103.47	3.87
Apigenin-7-glucoside	0.56	29.16	30.63	103.12	3.98
		58.32	54.98	101.89	2.88
		145.8	131.72	98.88	2.24
Isochlorogenic acid C	123.39	30.0	152.84	98.17	3.04
		60.0	185.91	102.53	2.36
		150.01	272.26	99.24	3.48
Luteolin	0.84	28.34	29.26	100.28	2.41
		56.68	55.10	96.97	2.32
		141.7	148.54	103.95	4.20
Quercetin	10.03	29.5	38.60	96.85	4.69
		59.01	67.48	97.36	2.55
		147.5	160.25	101.84	3.56
Apigenin	0.24	28.86	27.92	99.38	4.12
		57.72	56.93	98.22	3.15
		144.3	143.57	99.33	2.30
Casticin	2.94	27.3	29.03	97.03	3.40
		54.6	54.63	94.67	2.30
		136.5	142.14	101.98	2.65

### 2.5. Sample Analysis

The validated method was successfully applied for the identification and quantification of 29 target compounds in 13 batches of YHKGT. The contents of the investigated compounds, based on their respective calibration curves, are summarized in Table 4. There were great variations among the contents of 24 compounds in different batches of YHKGT with RSD values exceeding 10%. Among them, chlorogenic acid, isochlorogenic acid A and isochlorogenic acid C, sweroside and agnuside, and schaftoside and rutin were the major phenolic acids, iridoids and flavonoids respectively, which have been found to have antiviral, anti-inflammatory and immunomodulatory properties [27,28,29,30]. Therefore, these three types of compounds should be considered as important bioactive components of YHKGT, and their content variabilities could influence the quality and efficacy of YHKGT.

**Table 4:** Contents of 29 investigated compounds in 13 batches of YHKGT samples.

Samples	Content of Each Compound in 13 Batches of YHKGP Samples (mg/g)																												
No.	1 ^a^	2	3	4	5	6	7	8	9	10	11	12	13	14	15	16	17	18	19	20	21	22	23	24	25	26	27	28	29
1208010	0.1580	0.5075	0.0186	0.1248	2.9787	0.7077	0.0211	0.0102	0.6596	0.9358	0.2806	0.1604	0.0056	0.0040	0.1847	0.0131	0.0248	0.1229	0.0767	0.5631	0.0477	0.9640	0.0420	0.0029	1.1698	0.0171	0.0898	0.0051	0.0156
1209015	0.1636	0.4144	0.0214	0.0666	3.6644	0.5157	0.0287	0.0102	0.5304	0.4690	0.1894	0.0890	0.0050	0.0043	0.1984	0.0074	0.0268	0.1084	0.0729	0.4345	0.0419	1.4652	0.0285	0.0034	1.1410	0.0121	0.0771	0.0051	0.0155
1211023	0.1467	0.3324	0.0236	0.0857	3.3666	0.5195	0.0270	0.0101	0.4447	0.6714	0.2512	0.1423	0.0051	0.0039	0.1920	0.0091	0.0257	0.1049	0.0585	0.4418	0.0654	1.3217	0.0494	0.0029	1.0473	0.0081	0.0728	0.0043	0.0152
1301013	0.1652	0.5879	0.0268	0.0801	4.1785	0.7268	0.0480	0.0102	0.7163	0.5082	0.2279	0.1065	0.0056	0.0047	0.2528	0.0078	0.0234	0.1156	0.0959	0.6249	0.0527	1.5898	0.0426	0.0024	1.6397	0.0143	0.0748	0.0046	0.0179
1301014	0.1192	0.2926	0.0130	0.0602	2.2661	0.4318	0.0151	0.0099	0.3370	0.6574	0.2410	0.1313	0.0064	0.0039	0.1998	0.0074	0.0261	0.1069	0.0623	0.4129	0.0636	1.2913	0.0502	0.0021	0.7809	0.0025	0.0701	0.0040	0.0152
1302015	0.1367	0.5406	0.0196	0.0524	3.9758	0.7341	0.0317	0.0102	0.5921	0.5662	0.2158	0.0733	0.0050	0.0045	0.2423	0.0054	0.0221	0.1015	0.0808	0.6398	0.0425	1.5587	0.0335	0.0027	1.1480	0.0044	0.0699	0.0040	0.0172
1302016	0.1636	0.5390	0.0202	0.0701	3.4165	0.6541	0.0231	0.0101	0.5479	0.5190	0.1880	0.0759	0.0054	0.0051	0.1972	0.0066	0.0214	0.1153	0.0879	0.4715	0.0391	1.4159	0.0325	0.0042	1.0685	0.0106	0.0706	0.0041	0.0163
1302017	0.1685	0.6239	0.0274	0.0763	4.2379	0.7716	0.0371	0.0102	0.6172	0.5195	0.2091	0.1073	0.0050	0.0058	0.2065	0.0078	0.0145	0.1033	0.0809	0.6676	0.0420	1.6356	0.0257	0.0029	1.2690	0.0075	0.0698	0.0041	0.0161
1302018	0.1698	0.5489	0.0189	0.0566	3.6544	0.7684	0.0273	0.0101	0.6357	0.4679	0.2182	0.0666	0.0049	0.0042	0.1871	0.0091	0.0209	0.1042	0.0741	0.5110	0.0378	1.4709	0.0269	0.0042	1.0873	0.0069	0.0676	0.0040	0.0162
1304010	0.1693	0.5384	0.0304	0.0751	3.5976	0.6395	0.0235	0.0097	0.6612	0.5151	0.1797	0.0899	0.0048	0.0043	0.1979	0.0064	0.0231	0.1144	0.0799	0.4860	0.0359	1.4387	0.0299	0.0039	1.1153	0.0064	0.0668	0.0040	0.0161
1304023	0.1396	0.5508	0.0174	0.0532	4.0312	0.6471	0.0292	0.0101	0.6498	0.4772	0.2347	0.0739	0.0050	0.0042	0.2095	0.0073	0.0233	0.1046	0.0874	0.6507	0.0410	1.5325	0.0273	0.0017	1.2526	0.0083	0.0723	0.0037	0.0174
1304024	0.1717	0.5322	0.0175	0.1299	3.8052	0.7524	0.0267	0.0102	0.4648	0.8406	0.1597	0.1405	0.0049	0.0054	0.1987	0.0087	0.0174	0.1080	0.0719	0.5950	0.0484	1.4747	0.0317	0.0031	0.9447	0.0109	0.0780	0.0035	0.0155
1304025	0.1509	0.4240	0.0153	0.0941	2.7936	0.5772	0.0163	0.0101	0.4603	0.5562	0.2440	0.0815	0.0052	0.0037	0.1745	0.0071	0.0189	0.0907	0.0614	0.4857	0.0473	0.9683	0.0361	0.0015	0.7741	0.0094	0.0748	0.0035	0.0167
*Aver.*	0.1556	0.4948	0.0208	0.0789	3.5359	0.6497	0.0273	0.0101	0.5629	0.5926	0.2184	0.1029	0.0052	0.0045	0.2032	0.0080	0.0222	0.1077	0.0762	0.5373	0.0466	1.3944	0.0351	0.0029	1.1106	0.0091	0.0734	0.0042	0.0162
RSD (%)	9.89	19.22	23.23	30.35	15.63	16.19	30.31	1.34	18.80	23.86	14.62	29.13	8.07	13.59	10.73	22.69	15.55	7.16	13.94	16.12	19.06	14.68	22.97	28.85	19.13	41.86	7.84	12.08	5.03

^a^ The compound numbers are the same as in Figure 4.

## 3. Experimental Section

### 3.1. Standards, Reagents and Materials

Protocatechuic acid (**1**), neochlorogenic acid (**2**), protocatechualdehyde (**3**), 4-hydroxybenzoic acid (**4**), chlorogenic acid (**5**), cryptochlorogenin acid (**6**), caffeic acid (**7**), swertiamarin (**8**), sweroside (**9**), schaftoside (**10**), agnuside (**11**), isoschaftoside (**12**), flavosativaside (**13**), vitexin 2′′-rhamnoside (**14**), rutin (**15**), vitexin (**16**), hyperoside (**17**), isoquercitrin (**18**), luteoloside (**19**), isochlorogenic acid B (**20**), kaempferol-3-*O*-rutinoside (**21**), isochlorogenic acid A (**22**), astragalin (**23**), apigenin-7-glucoside (**24**), isochlorogenic acid C (**25**), luteolin (**26**), quercetin (**27**), apigenin (**28**), casticin (**29**), albiflorin (internal standard 1, **IS1**), liquiritin (internal standard 2, **IS2**) and 2-hydro xycinnamic acid (internal standard 3, **IS3**) were purchased from the Chinese National Institute for Control of Pharmaceutical and Biological Products (Beijing, China). The chemical structures of these compounds are shown in Figure 3. The purity of each reference standard was higher than 98% as determined by HPLC.

**Figure 4:**
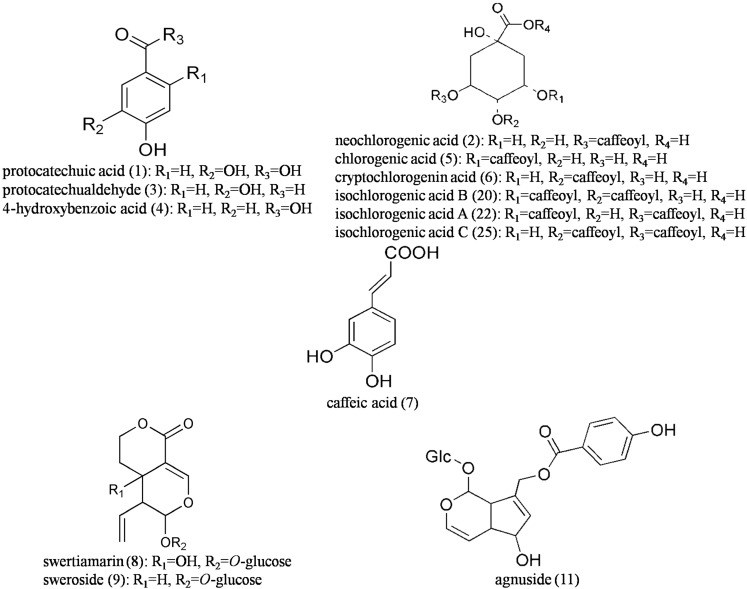

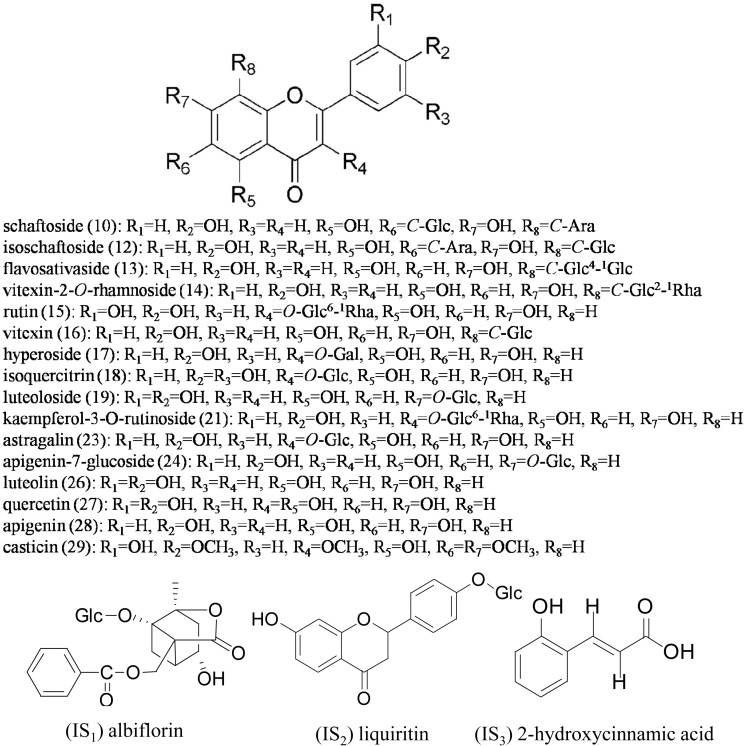
Chemical structures of the 29 investigated compounds and three internal standards.

Acetonitrile, methanol and formic acid (HPLC grade) for UPLC analysis were bought from Merck (Darmstadt, Germany). Deionized water was prepared using a Millipore Milli-Q purification system (Millipore, Bedford, MA, USA). Thirteen batches of Yin Hua Kang Gan Tablet (YHKGT) samples were obtained from Zhangzhou PienTzeHuang Pharmaceutical Co. Ltd. (Zhangzhou City, Fujian Province, China) and stored at 4 °C until analysis. Voucher specimens were deposited in the College of Pharmacy, Fujian University of Traditional Chinese Medicine.

### 3.2. Preparation of Standard Solution and Samples

Stock solutions of the 29 standards (approx. 1 mg/mL) were prepared individually by dissolving accurately weighted amount of standards in 70% methanol-water. An internal standards stock solution was also prepared in a concentration of 0.32 μg/mL for liquiritin, 0.25 μg/mL for 2-hydroxycinnamic acid and 0.81 μg/mL for albiflorin. Then a mixed solution containing all the 29 standards were prepared and serially diluted with 70% methanol-water (*v*/*v*) to obtain seven reference solutions with different concentrations used for plotting standard curves. All prepared solutions were stored at 4 °C before analysis. The 13 batches of YHKGT samples were ground to a fine powder. A powder sample (0.20 g) was accurately weighted and extracted with 100 mL of 70% methanol-water (*v*/*v*) in an ultrasonic bath (40 kHz, 500 w) for 30 min. Additional 70% methanol-water was added to make up the lost weight. The extracted solution was centrifuged at 150,000 rpm for 10 min, and the supernatant was obtained as sample solution. The internal standard working solution (500 μL) was added to 500 μL of the mixed standards solution or sample solution, then vortex blended and filtered through a 0.22 μm micropore membrane prior to injection. All the samples were stored at 4 °C before analysis.

### 3.3. Liquid Chromatography

Chromatographic analysis was performed on a Waters Acquity UPLC H-Class system (Milford, MA, USA) equipped with an online vacuum degasser, a binary pump, an autosampler, and a thermostated column compartment. Chromatographic separation was carried out at 45 °C on an Waters Cortecs UPLC C18 column (50 × 2.1 mm, 1.6 μm). The mobile phases consisted of acetonitrile (A) and 5% methanol and 0.1% formic acid in water (B). The gradient elution program was as follows: 3% A at 0–2 min, 3%–7% A at 2–4 min, 7%–13% A at 4–6 min, 13%–20% A at 6–8 min, 20%–55% A at 8–8.5 min, 55%–90% A at 8.5–9.5 min, 90% A at 9.5–9.95 min, 90%–3% A at 9.95–10 min, 3% A at 10–12 min. The flow rate was kept at 0.25 mL/min, and the injected sample volume was 2 μL.

### 3.4. Mass Spectrometry

Tandem mass spectrometry was performed on an Xevo TQ QqQ MS triple quadrupole mass spectrometer equipped with an electrospray ion source (ESI) (Waters). The MS spectra were acquired in multiple reaction monitoring (MRM) mode. Polarity switching electrospray ionization was applied. Argon was chosen as collision gas, nitrogen was chosen as nebulizer gas and heater gas. The MS conditions were optimized as follows: capillary voltage, 2.5 kV; source temperature, 200 °C; dwell time, 20 ms.

## 4. Conclusions

In this study, a UPLC-QqQ-MS method for the simultaneous determination of 29 major components in YHKGT has been developed and validated for the first time, which greatly improved its quality control. The polarity switching mode facilitated the detection of multiple types of constituents in YHKGT with different ionization responses. Compared with the current published HPLC method [13], this developed method enabled identification of target compounds with high selectivity by comparison with standards, high sensitivity and a rapid analysis was performed within 12 min. The results obtained in this work demonstrated that polarity switching in UPLC-QqQ-MS provides an unsuspected advantage for complex method development in the TCM analytical services industry.

## Supplementary Materials

Supplementary materials can be accessed at: http://www.mdpi.com/1420-3049/20/07/12209/s1.
