# Supplementary material for: Qualitative and Quantitative Analysis of Phenolic Acids, Flavonoids and Iridoid Glycosides in Yinhua Kanggan Tablet by UPLC-QqQ-MS/MS
Source: Molecules. 2015 Jul 3;20(7):12209–28. doi: 10.3390/molecules200712209 (PMC6331852; doi:10.3390/molecules200712209)
Supplement: Supplementary file 1 [file molecules-20-12209-s001.pdf]

Supplementary Material

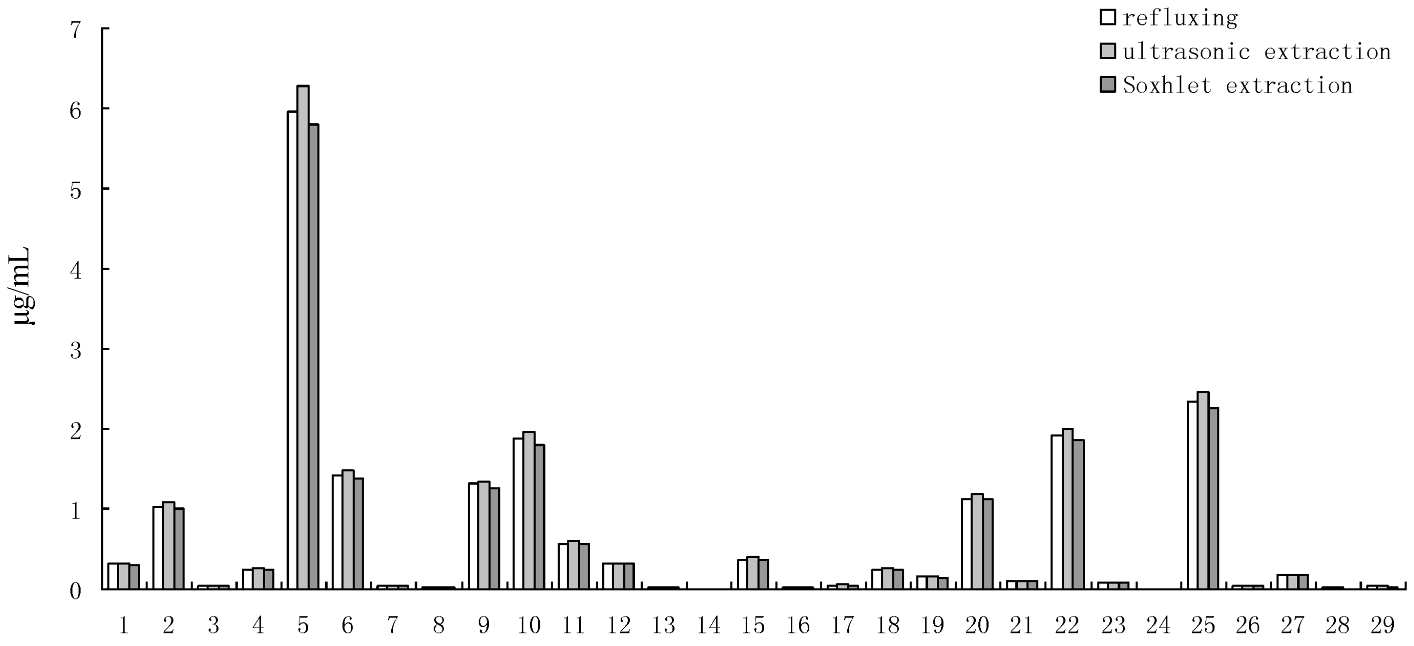

Figure S1. Different extraction method in extracting typical compounds **1–29** using 70% aqueous methanol as extraction solvent.

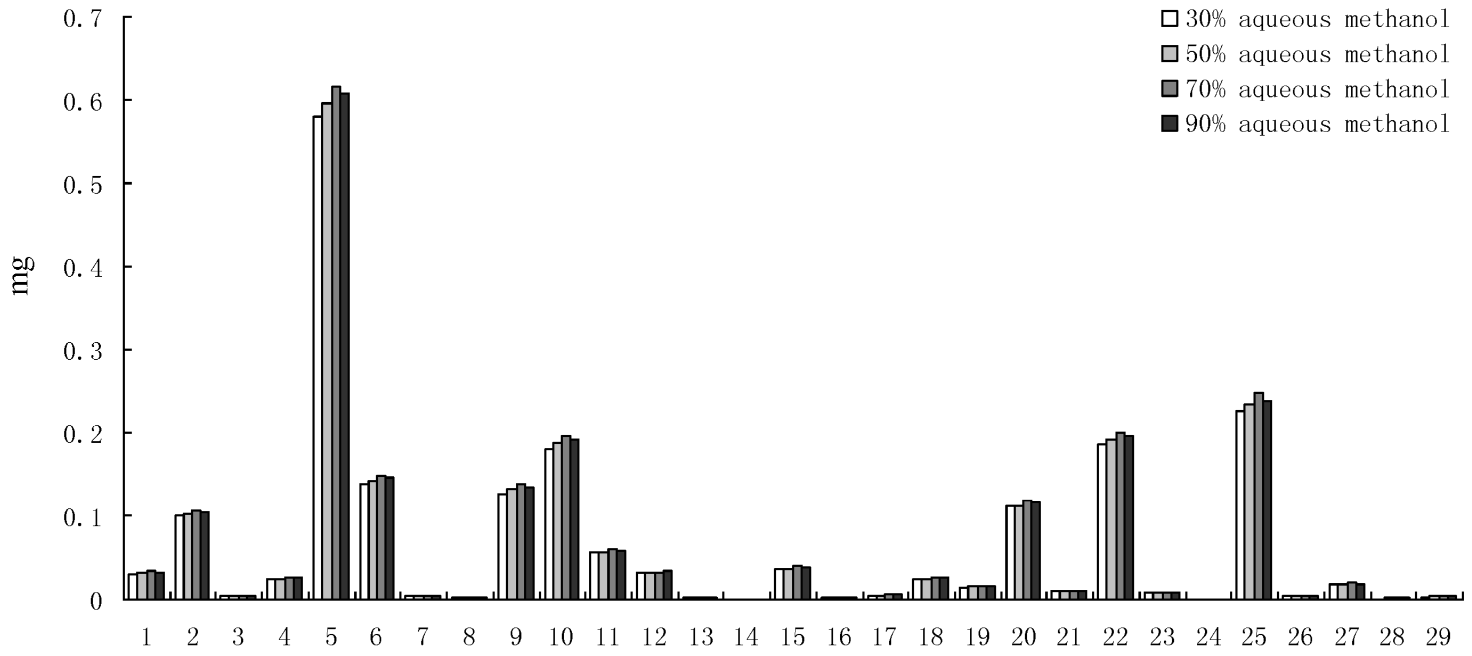

Figure S2. Different concentrations of methanol-water solution (30%, 50%, 70% and 90%) in extracting typical compounds **1**–**29**.

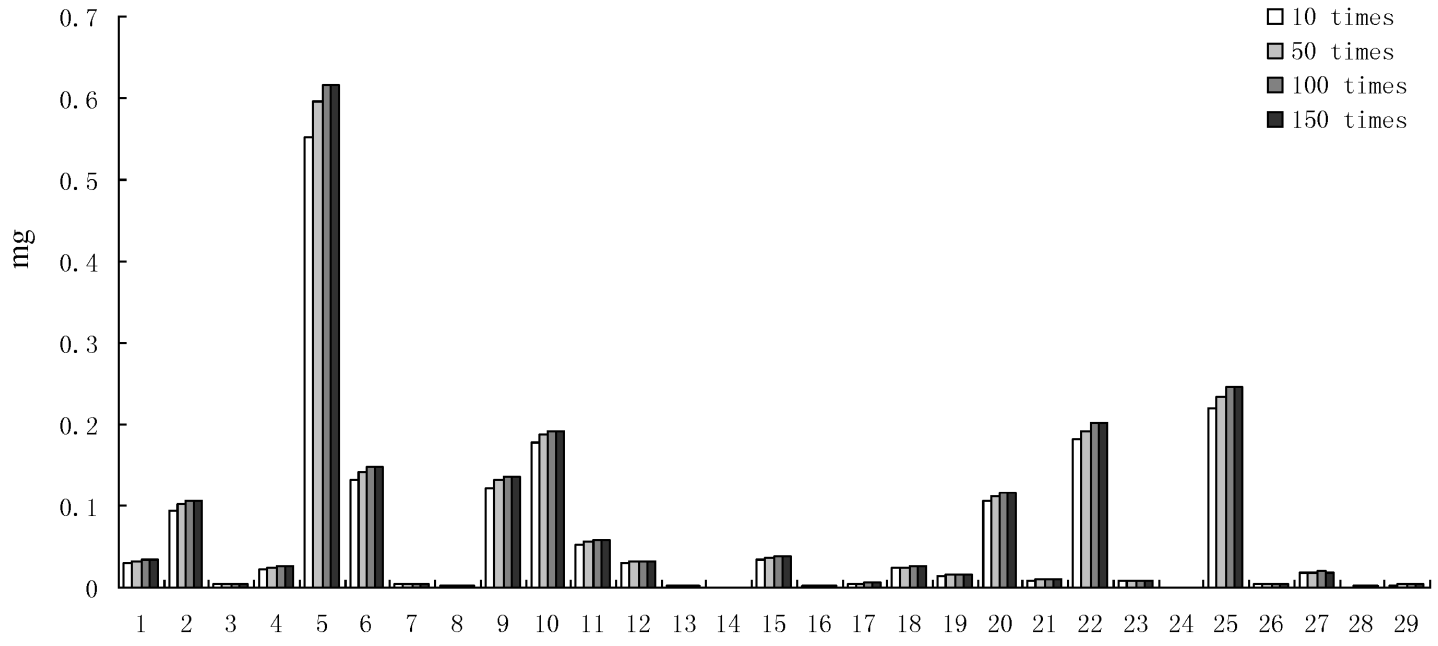

Figure S3. Different extraction time (15, 30, 45 and 60 min) in extracting typical compounds **1**–**29**.

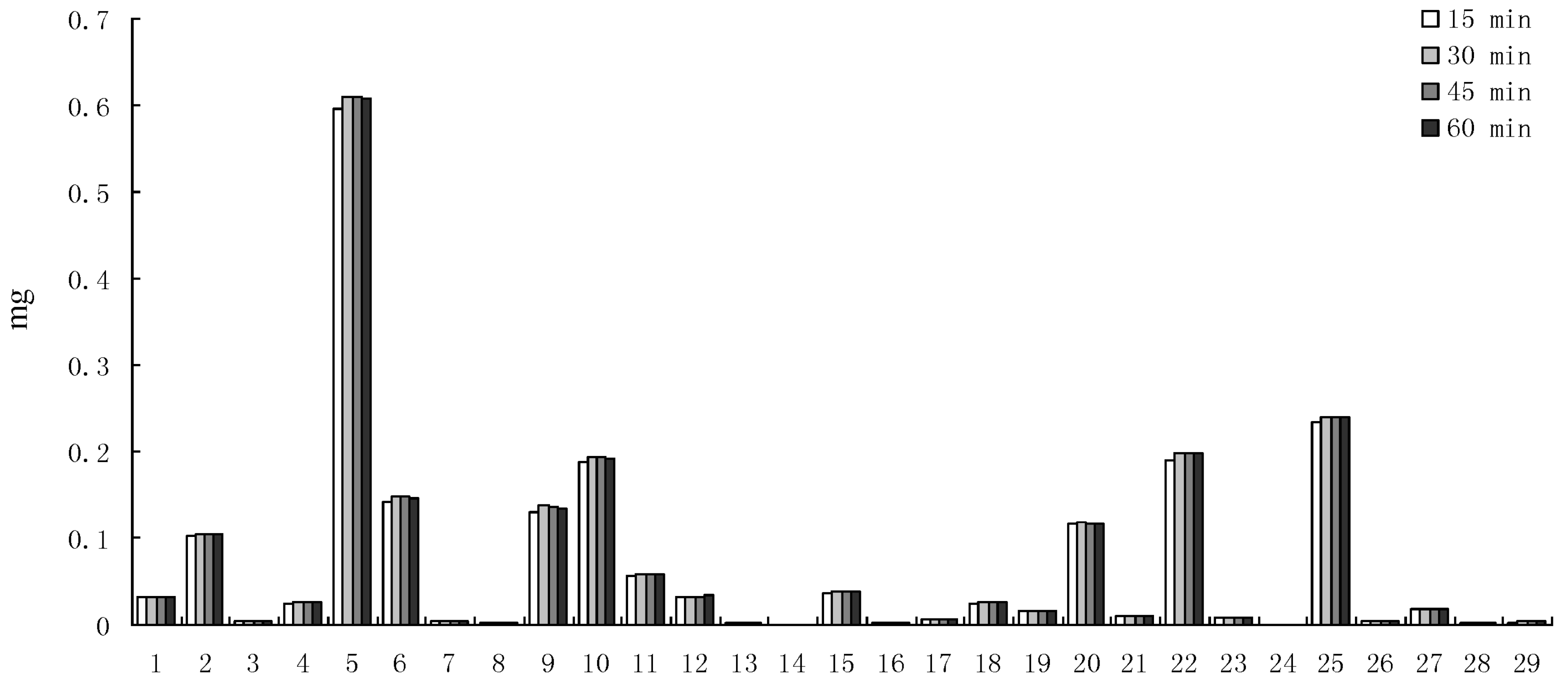

Figure S4. Different solvent volume (10, 50, 100 and 150 times) in extracting typical compounds **1**–**29**.

Table S1. Mass spectra of the standards.

No.	Analytes	Formula	Structure	Mass Spectrum	Quasi-Molecular Ion and Product Ions
1	Protocatechuic acid	$C_7H_6O_4$	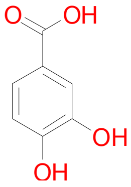	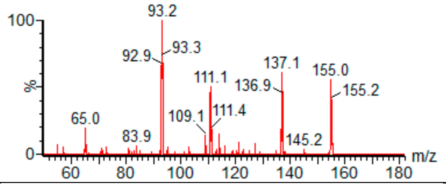	$[M + H]^+$ 155<br>$[M + H - H_2O]^+$ 137<br>$[M + H - CO_2]^+$ 111<br>$[M + H - H_2O - CO_2]^+$ 93
2	Neochlorogenic acid	$C_{16}H_{18}O_9$	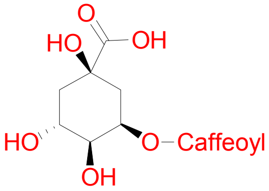	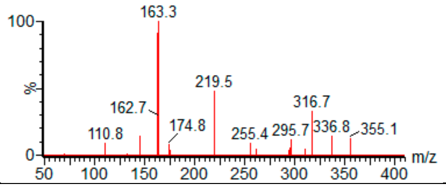	$[M + H]^+$ 355<br>$[M + H - H_2O]^+$ 337<br>$[M + H - \text{quinic acid}]^+$ 163<br>$[M + H - \text{quinic acid} - H_2O]^+$ 145
3	Protocatechualdehyde	$C_7H_6O_3$	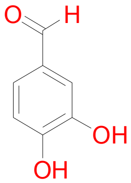	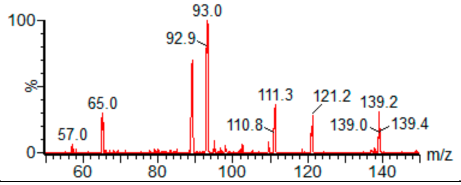	$[M + H]^+$ 139<br>$[M + H - H_2O]^+$ 121<br>$[M + H - CO]^+$ 111<br>$[M + H - H_2O - CO]^+$ 93
4	*p*-hydroxybenzoic acid	$C_7H_6O_3$	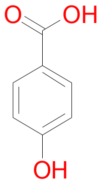	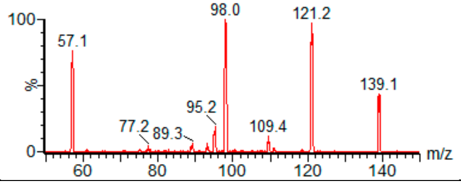	$[M + H]^+$ 139<br>$[M + H - H_2O]^+$ 121<br>$[M + H - CO_2]^+$ 95<br>$[M + H - H_2O - CO_2]^+$ 77
5	Chlorogenic acid	$C_{16}H_{18}O_9$	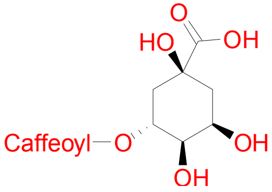	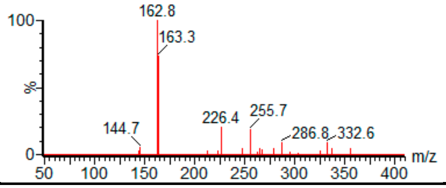	$[M + H]^+$ 355<br>$[M + H - H_2O]^+$ 337<br>$[M + H - \text{quinic acid}]^+$ 163<br>$[M + H - \text{quinic acid} - H_2O]^+$ 145

Table S1. *Cont.*

No.	Analytes	Formula	Structure	Mass Spectrum	Quasi-Molecular Ion and Product Ions
6	Cryptochlorogenin acid	$C_{16}H_{18}O_9$	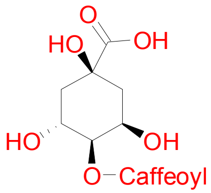	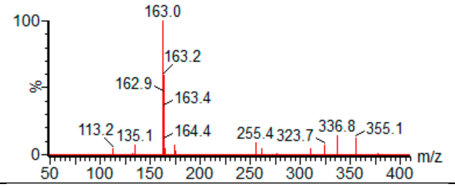	$[M + H]^+$ 355<br>$[M + H - H_2O]^+$ 337<br>$[M + H -$ quinic acid$]^+$ 163<br>$[M + H -$ quinic acid $- CO]^+$ 135
7	Caffeic acid	$C_9H_8O_4$	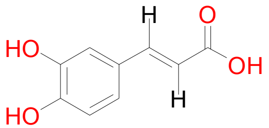	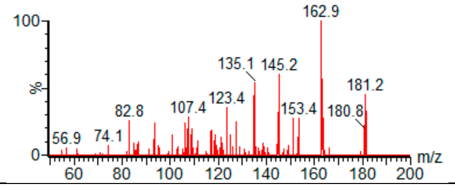	$[M + H]^+$ 181<br>$[M + H - H_2O]^+$ 163<br>$[M + H - CO_2]^+$ 135<br>$[M + H - H_2O - CO_2]^+$ 123
8	Swertiamarin	$C_{16}H_{22}O_{10}$	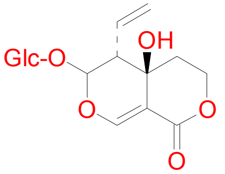	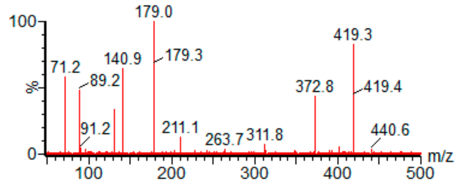	$[M - H + HCOOH]^-$ 419<br>$[M - H]^-$ 373<br>$[M - H - H_2O]^-$ 355<br>$[M - H - H_2O - CO_2]^-$ 311<br>$[M - H -$ Glc$]^-$ 211<br>$[$glucose $- H]^-$ 179
9	Sweroside	$C_{16}H_{22}O_9$	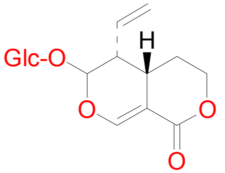	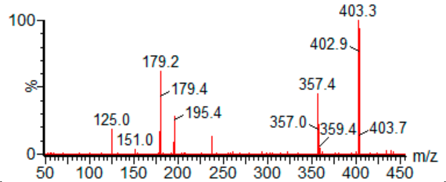	$[M - H + HCOOH]^-$ 403<br>$[M - H]^-$ 357<br>$[M - H - Glc]^-$ 195<br>$[$glucose $- H]^-$ 179<br>$[M - H -$ Glc $- C_4H_6O]^+$ 125
10	Schaftoside	$C_{26}H_{28}O_{14}$	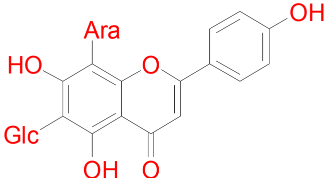	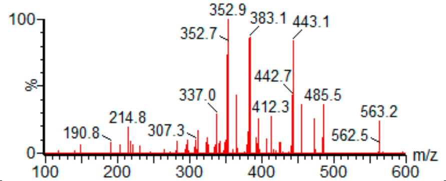	$[M - H]^-$ 563<br>$[M - H - H_2O]^-$ 545<br>$[M - H - 3CH_2O]^-$ 473<br>$[M - H - 4CH_2O]^-$ 443<br>$[M - H - 6CH_2O]^-$ 383<br>$[M - H - 7CH_2O]^-$ 353

Table S1. *Cont.*

No.	Analytes	Formula	Structure	Mass Spectrum	Quasi-Molecular Ion and Product Ions
11	Agnuside	$C_{22}H_{26}O_{11}$	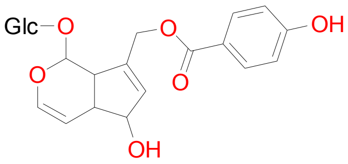	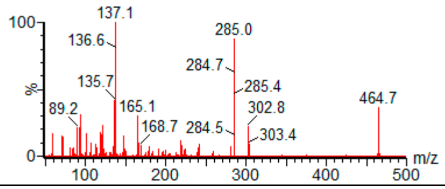	$[M-H]^-$ 465<br>$[M-H-Glc]^-$ 303<br>$[M-H-Glc-H_2O]^-$ 285<br>[M − H − Glc-*p*-hydroxybenzoic acid]$^-$ 165<br>[*p*-hydroxybenzoic acid− H]$^-$ 137
12	Isoschaftoside	$C_{26}H_{28}O_{14}$	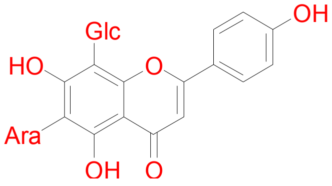	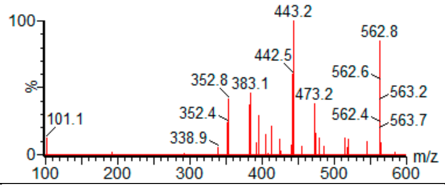	$[M-H]^-$ 563<br>$[M-H-3CH_2O]^-$ 473<br>$[M-H-4CH_2O]^-$ 443<br>$[M-H-6CH_2O]^-$ 383<br>$[M-H-7CH_2O]^-$ 353
13	Flavosativaside	$C_{27}H_{30}O_{15}$	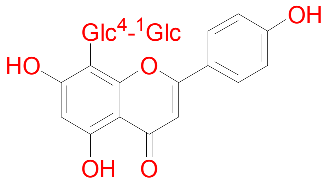	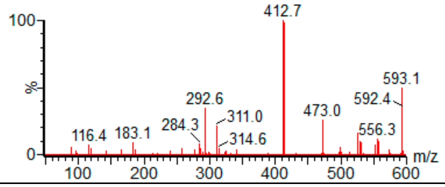	$[M-H]^-$ 593<br>$[M-H-4CH_2O]^-$ 473<br>$[M-H-Glc]^-$ 413<br>$[M-H-Glc-4CH_2O]^-$ 293
14	Vitexin 2″-rhamnoside	$C_{27}H_{30}O_{14}$	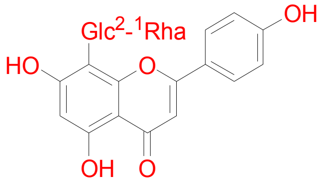	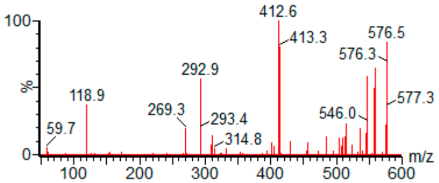	$[M-H]^-$ 577<br>$[M-H-H_2O]^-$ 559<br>$[M-H-CH_2O]^-$ 547<br>$[M-H-Rha]^-$ 413<br>$[M-H-Rha-4CH_2O]^-$ 293
15	Rutin	$C_{27}H_{30}O_{16}$	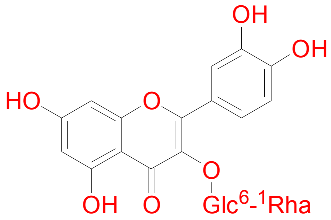	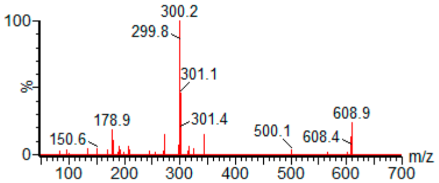	$[M-H]^-$ 609<br>$[M-H-Glc-Rha]^-$ 300<br>$[M-H-Glc-Rha-CH_2O]^-$ 271<br>[aglycone $A^{1,2}$ − H]$^-$ 179<br>[aglycone $A^{1,3}$ − H]$^-$ 151

Table S1. *Cont.*

No.	Analytes	Formula	Structure	Mass Spectrum	Quasi-Molecular Ion and Product Ions
16	Vitexin	$C_{21}H_{20}O_{10}$	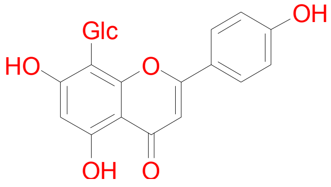	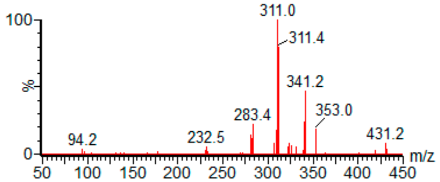	$[M-H]^-$ 431<br>$[M-H-3CH_2O]^-$ 341<br>$[M-H-3CH_2O-H_2O]^-$ 323<br>$[M-H-4CH_2O]^-$ 311<br>$[M-H-4CH_2O-CO]^-$ 283
17	Hyperoside	$C_{21}H_{20}O_{12}$	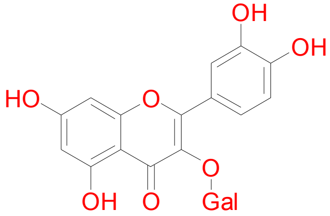	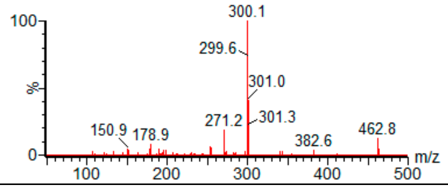	$[M-H]^-$ 463<br>$[M-H-Gal]^-$ 300<br>$[M-H-Gal-CH_2O]^-$ 271<br>$[aglycone\ A^{1,2}-H]^-$ 179<br>$[aglycone\ A^{1,3}-H]^-$ 151
18	Isoquercitrin	$C_{21}H_{20}O_{12}$	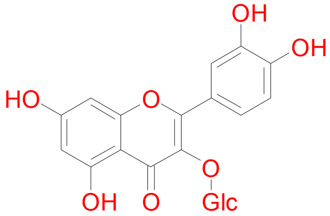	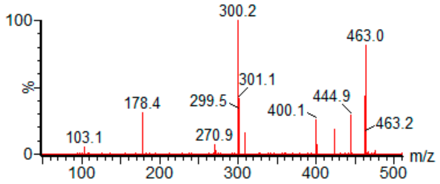	$[M-H]^-$ 463<br>$[M-H-H_2O]^-$ 445<br>$[M-H-H_2O-CO_2]^-$ 401<br>$[M-H-Glc]^-$ 300<br>$[M-H-Glc-CH_2O]^-$ 271<br>$[aglycone\ A^{1,2}-H]^-$ 179
19	Luteoloside	$C_{21}H_{20}O_{11}$	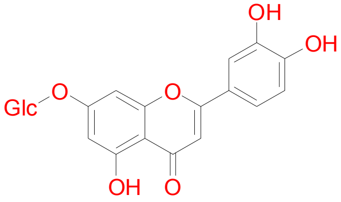	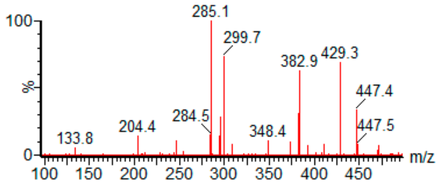	$[M-H]^-$ 447<br>$[M-H-H_2O]^-$ 429<br>$[M-H-H_2O-CH_2O_2]^-$ 383<br>$[M-H-Glc]^-$ 285

Table S1. *Cont.*

No.	Analytes	Formula	Structure	Mass Spectrum	Quasi-Molecular Ion and Product Ions
20	Isochlorogenic acid B	$C_{25}H_{24}O_{12}$	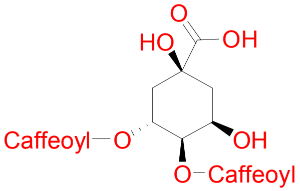	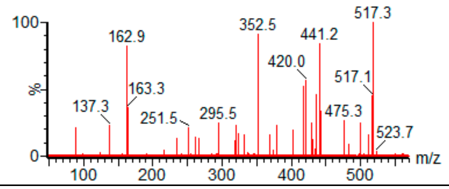	$[M + H]^+$ 517<br>$[M + H - H_2O]^+$ 499<br>$[M + H - C_2H_2O]^+$ 475<br>$[M + H - 2CO_2]^+$ 429<br>$[M + H -$ Caffeoyl $-$ quinic acid$]^+$ 163
21	Kaempferol-3-*O*-rutinoside	$C_{27}H_{30}O_{15}$	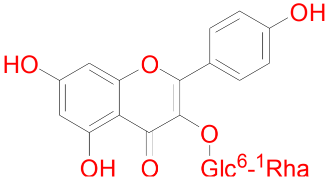	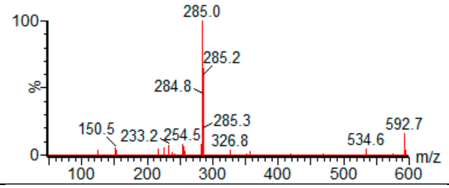	$[M - H]^-$ 593<br>$[M - H - CH_2O - CO]^-$ 535<br>$[M - H - Glc]^-$ 285<br>$[M - H - Glc - CH_2O]^-$ 255<br>[aglycone $A^{1,3} - H]^-$ 151
22	Isochlorogenic acid A	$C_{25}H_{24}O_{12}$	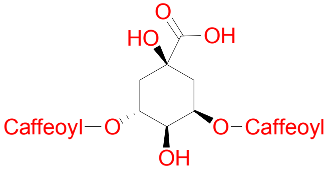	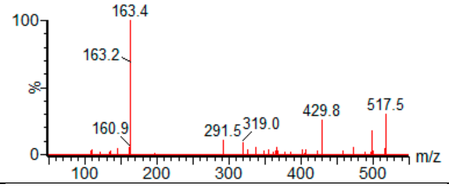	$[M + H]^+$ 517<br>$[M + H - H_2O]^+$ 499<br>$[M + H - 2CO_2]^+$ 429<br>$[M + H -$ Caffeoyl $-$ quinic acid$]^+$ 163
23	Astragalin	$C_{21}H_{20}O_{11}$	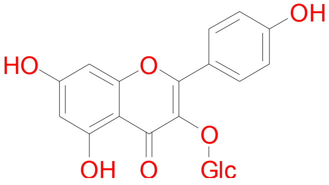	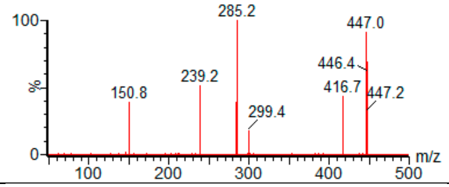	$[M - H]^-$ 447<br>$[M - H - CH_2O]^-$ 417<br>$[M - H - Glc]^-$ 285<br>$[M - H - Glc - CO - H_2O]^+$ 239<br>[aglycone $A^{1,3} - H]^-$ 151
24	Apigenin-7-glucoside	$C_{21}H_{20}O_{10}$	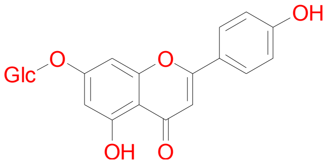	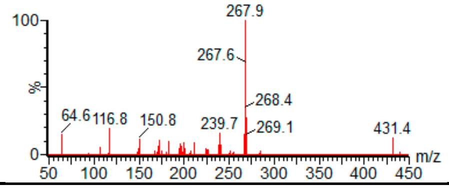	$[M - H]^-$ 431<br>$[M - H - Glc]^-$ 267<br>$[M - H - Glc - CO]^-$ 239<br>[aglycone $A^{1,3} - H]^-$ 151<br>[aglycone $B^{1,3} - H]^-$ 117

Table S1. *Cont.*

No.	Analytes	Formula	Structure	Mass Spectrum	Quasi-Molecular Ion and Product Ions
25	Isochlorogenic acid C	$C_{25}H_{24}O_{12}$	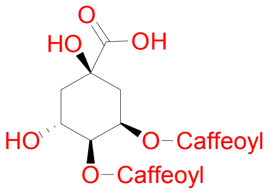	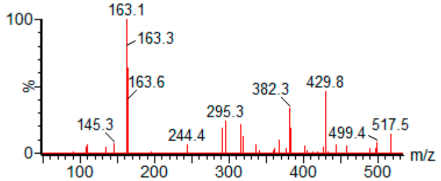	$[M + H]^+$ 517<br>$[M + H - H_2O]^+$ 499<br>$[M + H - 2CO_2]^+$ 429<br>$[M + H - Caffeoyl - quinic\ acid]^+$ 163
26	Luteolin	$C_{15}H_{10}O_6$	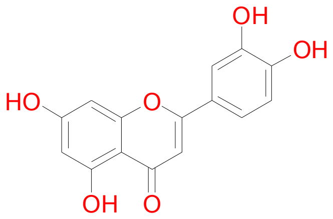	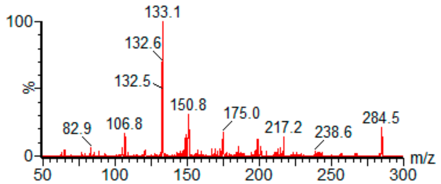	$[M - H]^-$ 285<br>$[M - H - H_2O - CH_2O]^-$ 237<br>$[M - H - B\ ring(C_6H_6O_2)]^-$ 175<br>$[A^{1,3}- H]^-$ 151<br>$[B^{1,3}- H]^-$ 133
27	Quercetin	$C_{15}H_{10}O_7$	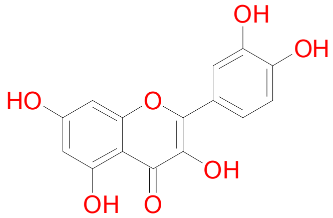	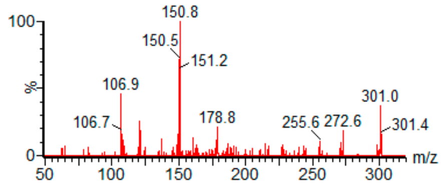	$[M - H]^-$ 301<br>$[M - H - CO]^-$ 273<br>$[M - H - CH_2O_2]^-$ 255<br>$[A^{1,3} - H]^-$ 151<br>$[B^{1,3} - H]^-$ 133
28	Apigenin	$C_{15}H_{10}O_5$	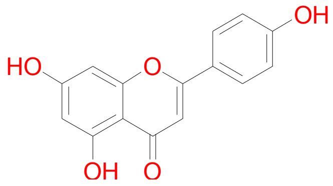	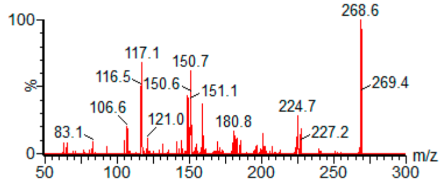	$[M - H]^-$ 267<br>$[M - H - C_2H_2O]^-$ 225<br>$[A^{1,3} - H]^-$ 151<br>$[B^{1,3} - H]^-$ 117
29	Casticin	$C_{19}H_{18}O_8$	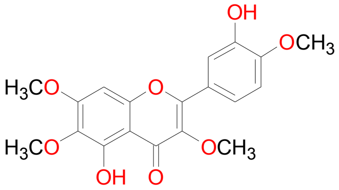	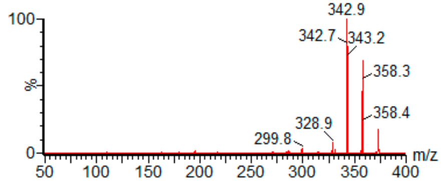	$[M - H]^-$ 373<br>$[M - H - CH_3]^-$ 358<br>$[M - H - 2CH_3]^-$ 343<br>$[M - H - 3CH_3]^-$ 328<br>$[M - H - 3CH_3 - CO]^-$ 300

Table S1. *Cont.*

No.	Analytes	Formula	Structure	Mass Spectrum	Quasi-Molecular Ion and Product Ions
30	Albiflorin (IS1)	$C_{23}H_{28}O_{11}$	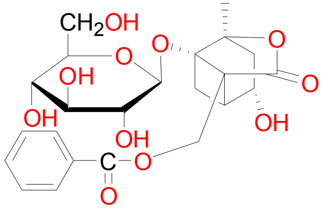	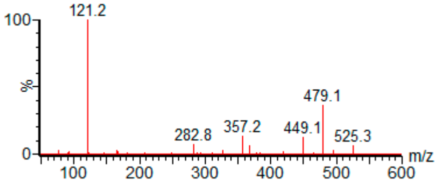	$[M-H+HCOOH]^-$ 525<br>$[M-H]^-$ 479<br>$[M-H-CH_2O]^-$ 449<br>$[M+H-CH_2O-\text{benzoic acid}]^-$ 357<br>$[\text{benzoic acid}-H]^-$ 121
31	Liquiritin (IS2)	$C_{21}H_{22}O_9$	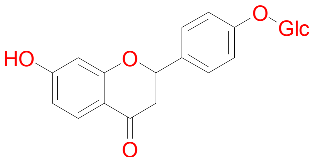	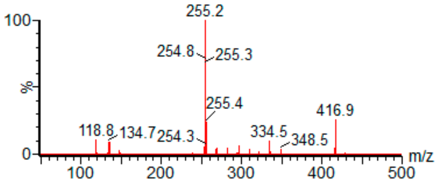	$[M-H]^-$ 417<br>$[M-H-Glc]^-$ 255<br>$[\text{aglycone } A^{1,3}-H]^-$ 135<br>$[\text{aglycone } B^{1,3}-H]^-$ 121
32	2-hydroxycinnamic acid (IS3)	$C_9H_8O_3$	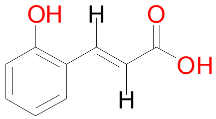	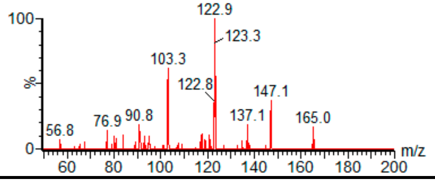	$[M+H]^+$ 165<br>$[M+H-H_2O]^+$ 147<br>$[M+H-CO]^+$ 137<br>$[M+H-C_2H_2O]^+$ 123<br>$[M+H-CO_2]^+$ 121<br>$[M+H-H_2O-CO_2]^+$ 103

Table S2. Regression equation, LOD and LOQ of 29 investigated compounds.

Compounds	Linear Regression Data			LOD (ng/mL)	LOQ (ng/mL)
	Regression Equation	*r*	Test Range (ng/mL)		
Protocatechuic acid	$y = 1.7855x - 0.0055$	0.9984	12.08–1836.9	1.37	3.86
Neochlorogenic acid	$y = 3.1969x - 0.0023$	0.9989	10.99–2463.1	0.65	1.78
Protocatechualdehyde	$y = 1.9264x - 0.0223$	0.9991	10.99–1986.9	1.08	4.73
p-hydroxybenzoic acid	$y = 0.8433x - 0.0125$	0.9982	45.98–1659.6	4.99	14.87
Chlorogenic acid	$y = 7.2982x - 0.0103$	0.9994	40.99–5470.3	0.34	1.49
Cryptochlorogenin acid	$y = 3.7792x - 0.0059$	0.9990	31.97–2451.8	0.55	1.97
Caffeic acid	$y = 3.1469x - 0.0031$	0.9991	22.85–1675.1	3.45	8.71
Swertiamarin	$y = 3.6247x + 0.0111$	0.9996	10.95–1502.9	0.07	0.65
Sweroside	$y = 0.6192x + 0.0017$	0.9994	19.78–2447.0	0.86	3.93
Schaftoside	$y = 0.5509x - 0.0024$	0.9987	21.98–2462.8	0.60	1.26
Agnuside	$y = 3.5786x + 0.0006$	0.9989	10.98–1974.8	0.08	0.55
Isoschaftoside	$y = 0.6451x + 0.0017$	0.9997	10.98–1859.7	0.29	0.89
Flavosativaside	$y = 3.5843x - 0.0139$	0.9994	2.963–1623.1	0.08	0.24
Vitexin 2''-rhamnoside	$y = 3.0184x - 0.0084$	0.9991	2.980–1790.3	0.14	0.66
Rutin	$y = 3.5643x - 0.0217$	0.9988	10.02–1881.3	0.03	0.16
Vitexin	$y = 10.876x - 0.0060$	0.9991	1.370–2683.5	0.05	0.24
Hyperoside	$y = 3.8665x - 0.0036$	0.9998	10.03–1882.5	0.14	0.35
Isoquercitrin	$y = 6.4179x - 0.0070$	0.9997	10.99–1768.4	0.17	0.43
Luteoloside	$y = 9.8992x + 0.0547$	0.9991	10.98–1650.2	0.15	0.45
Isochlorogenic acid B	$y = 6.1652x - 0.0036$	0.9990	10.92–2365.5	3.14	1.47
Kaempferol-3-*O*-rutinoside	$y = 3.8675x + 0.0276$	0.9993	11.0–1877.83	0.06	0.29
Isochlorogenic acid A	$y = 5.5711x - 0.0083$	0.9986	30.98–2756.5	0.32	1.28
Astragalin	$y = 3.7077x + 0.0152$	0.9991	10.99–1666.0	0.19	0.59
Apigenin-7-glucoside	$y = 17.759x + 0.0170$	0.9989	0.972–1483.6	0.15	0.40
Isochlorogenic acid C	$y = 5.7113x - 0.0047$	0.9990	21.00–2877.8	0.25	1.37
Luteolin	$y = 10.165x + 0.0063$	0.9992	1.945–1596.1	0.31	0.92
Quercetin	$y = 5.0424x - 0.0361$	0.9988	12.95–1753.4	3.45	10.4
Apigenin	$y = 14.771x - 0.0055$	0.9996	2.962–1478.6	0.22	0.41
Casticin	$y = 14.554x - 0.0046$	0.9991	10.91–1900.9	0.18	0.32
